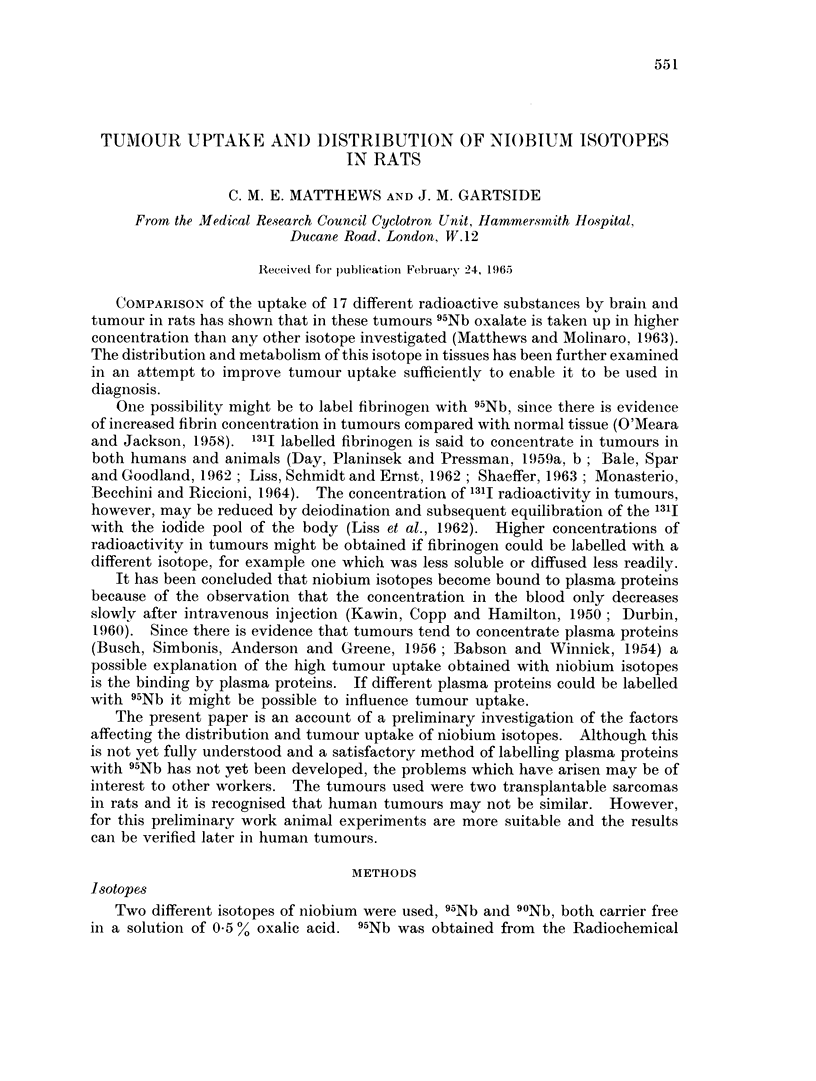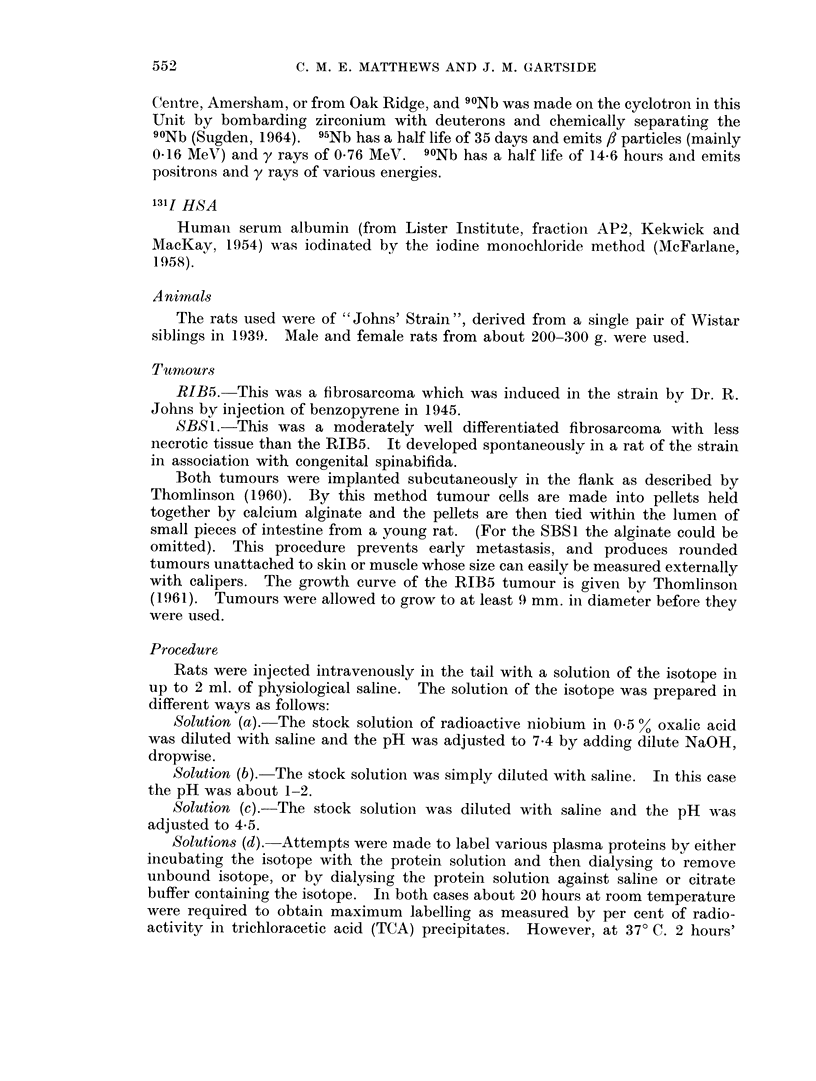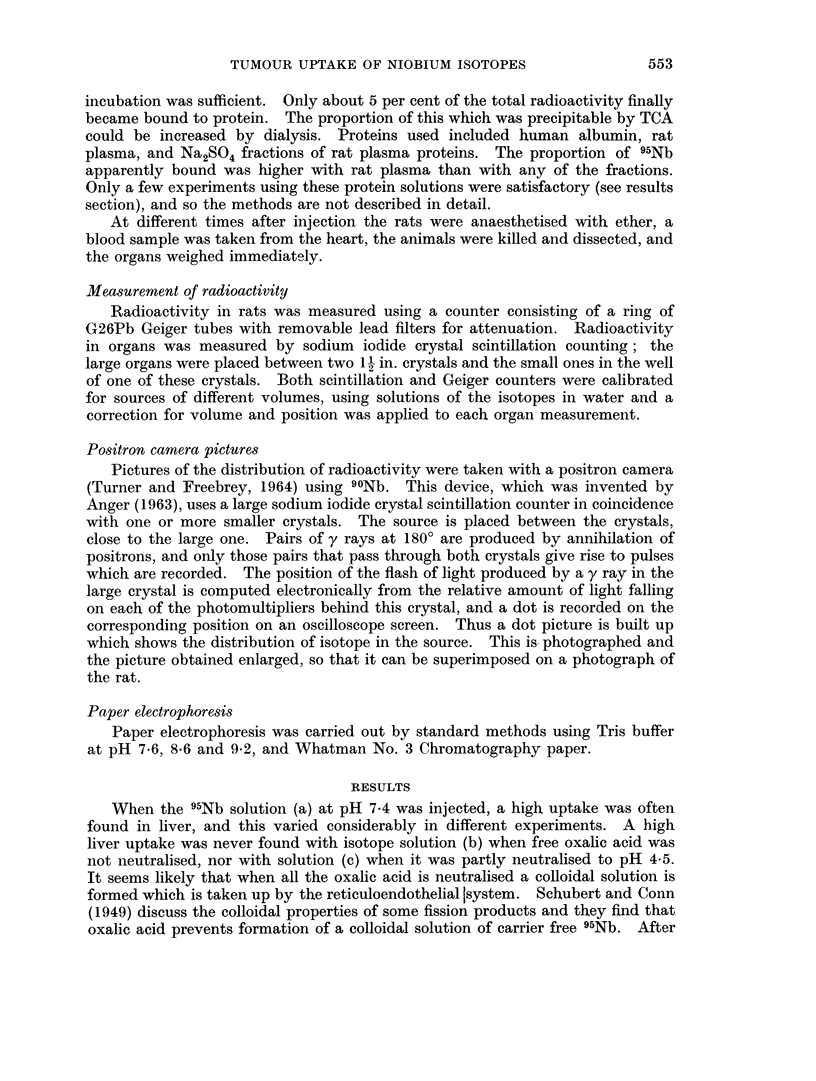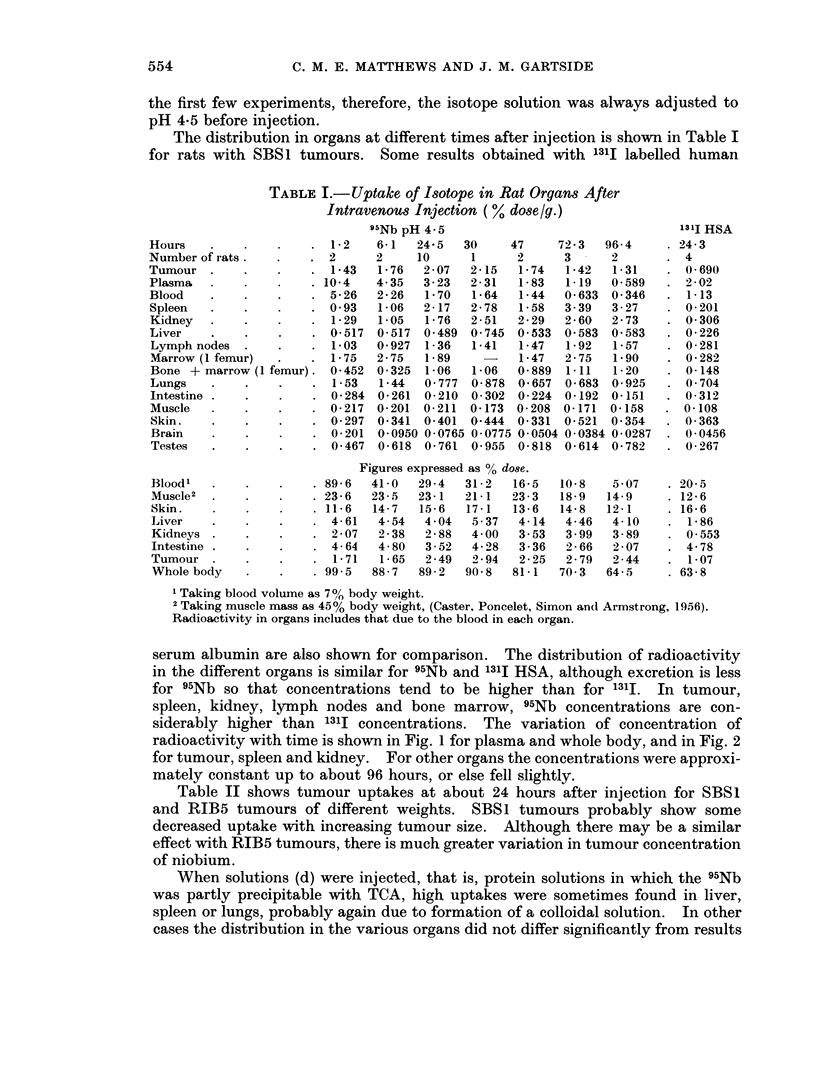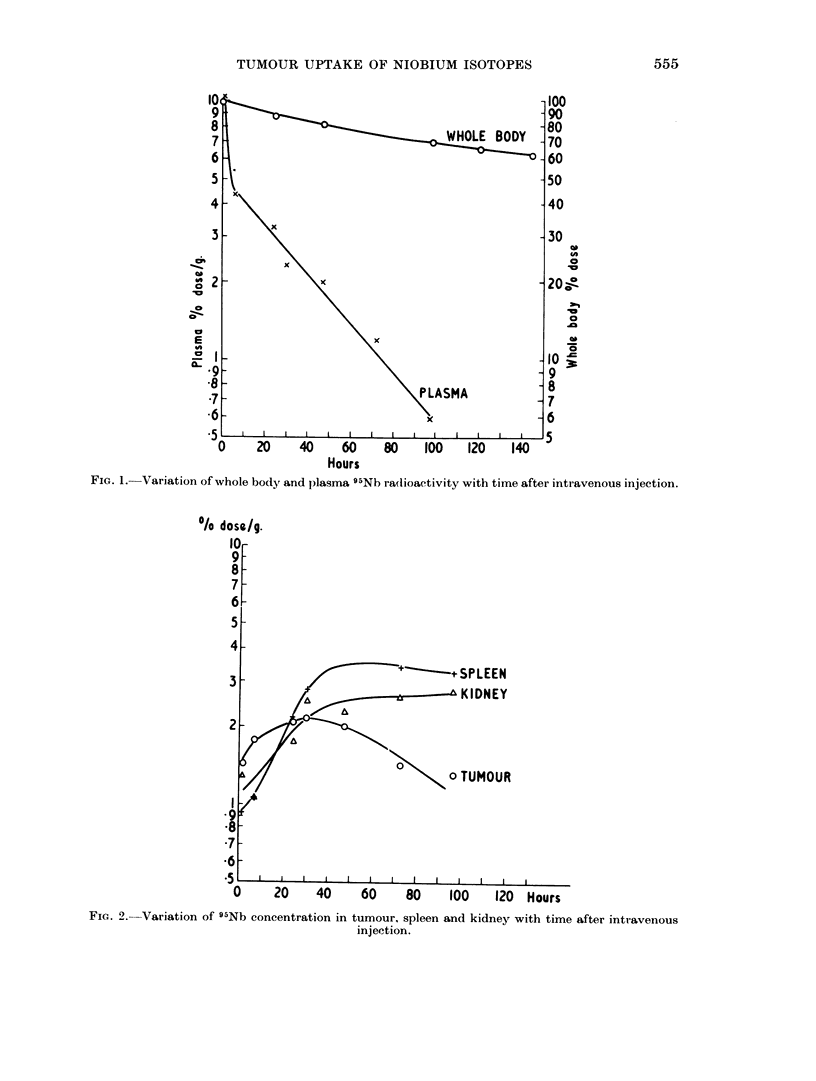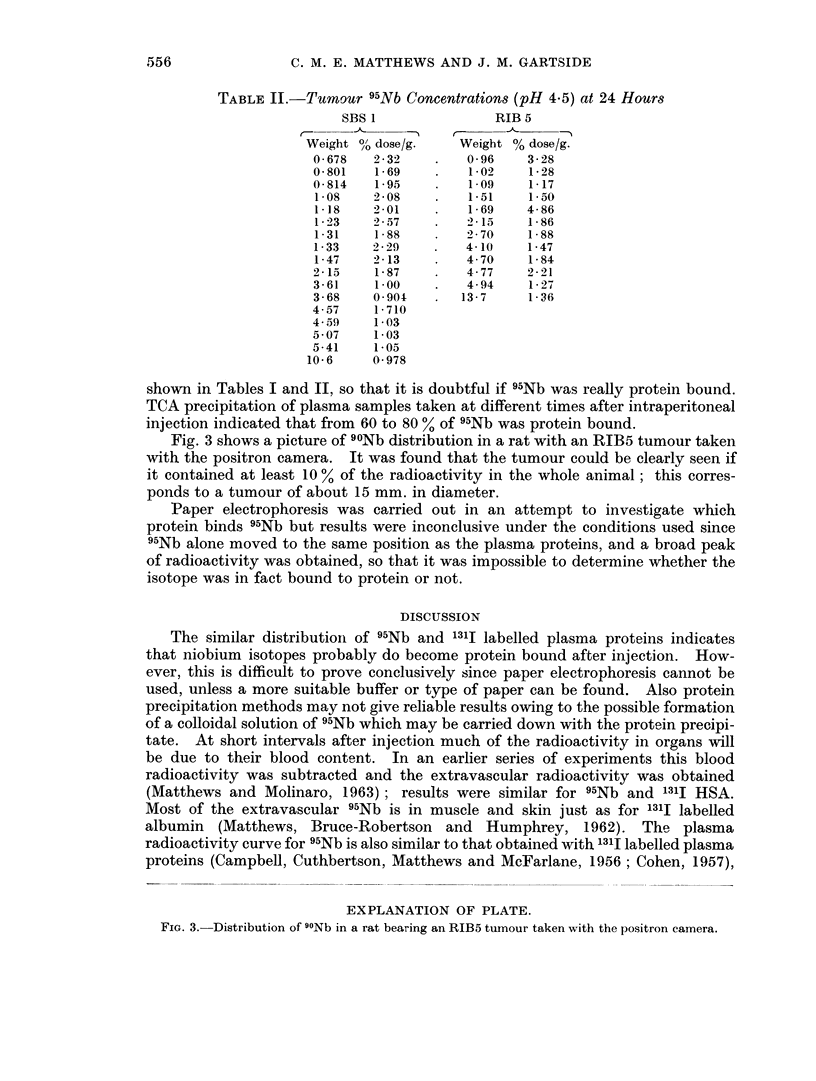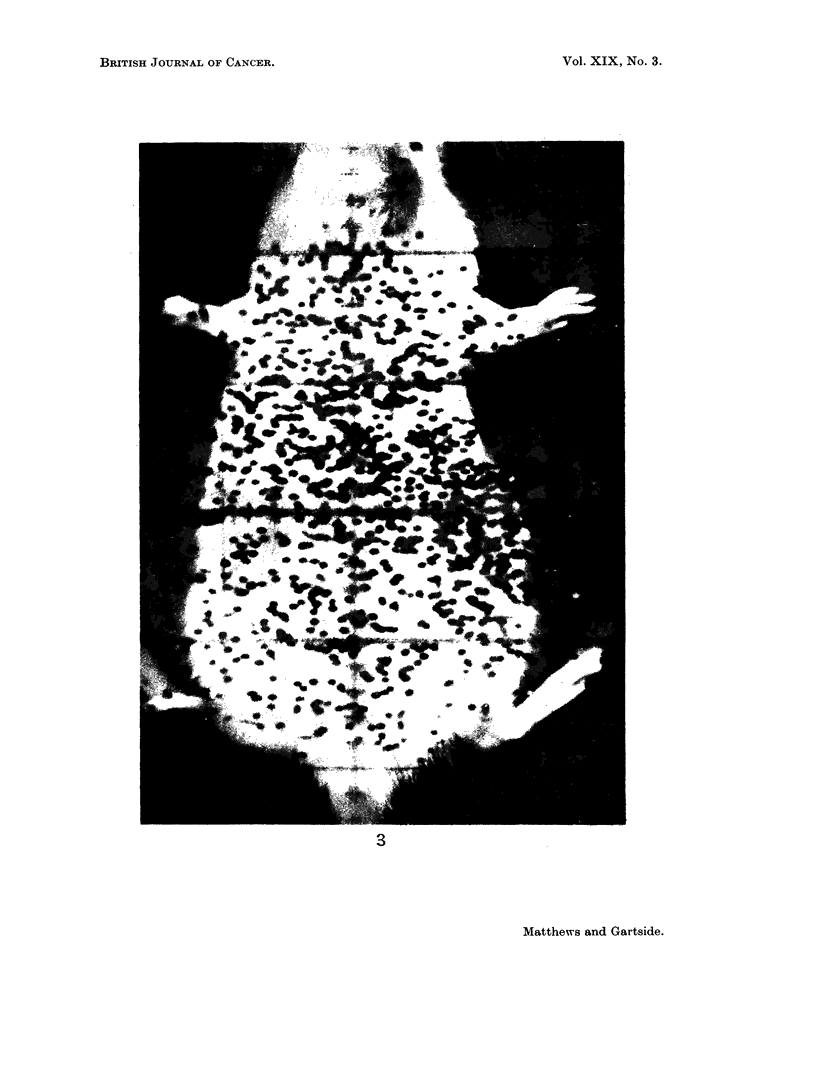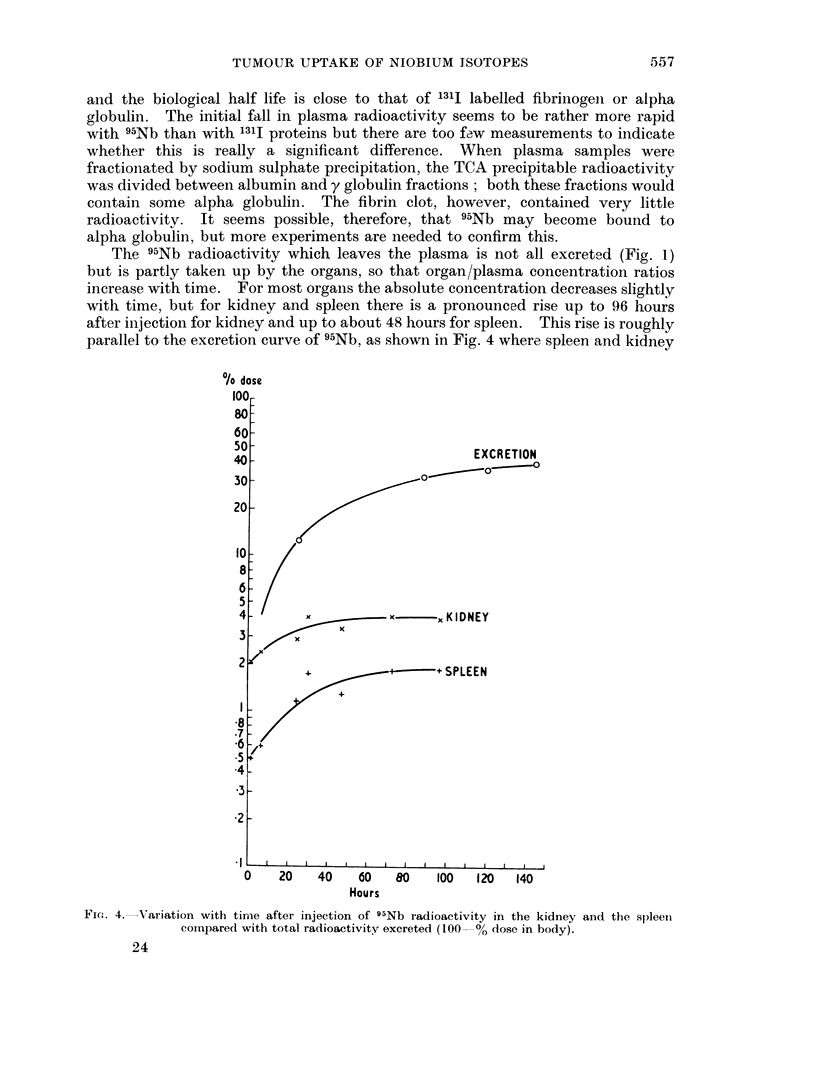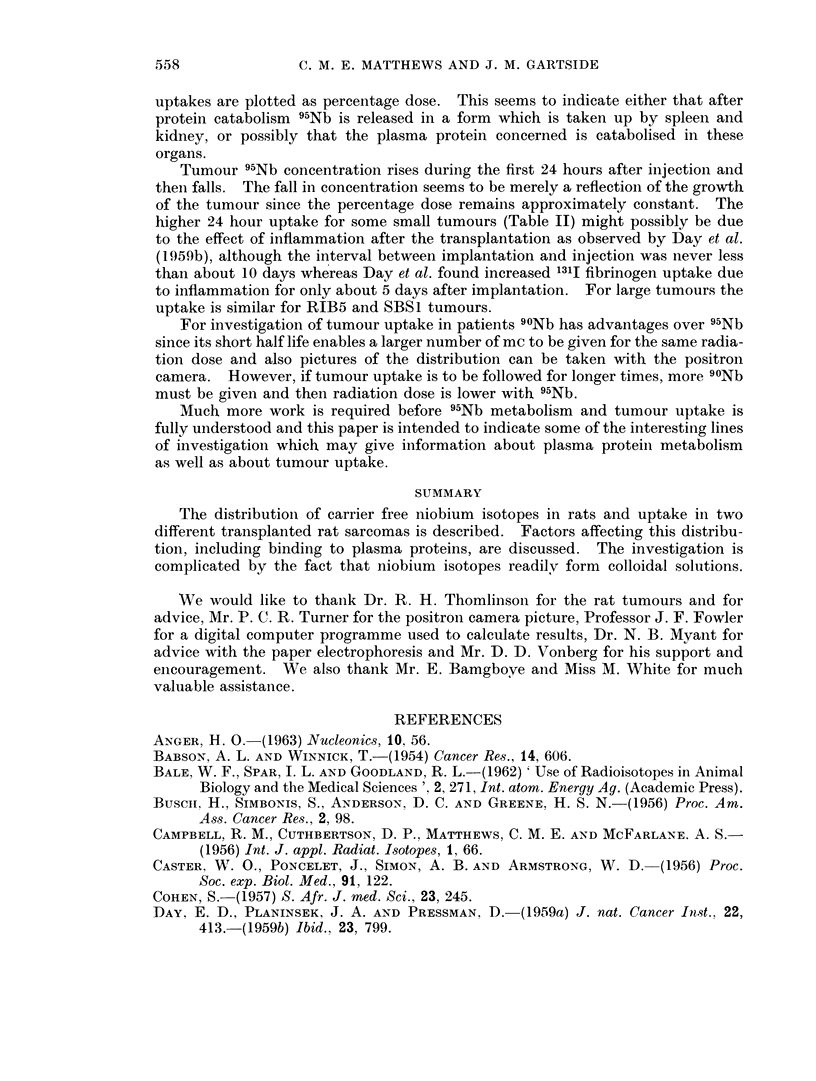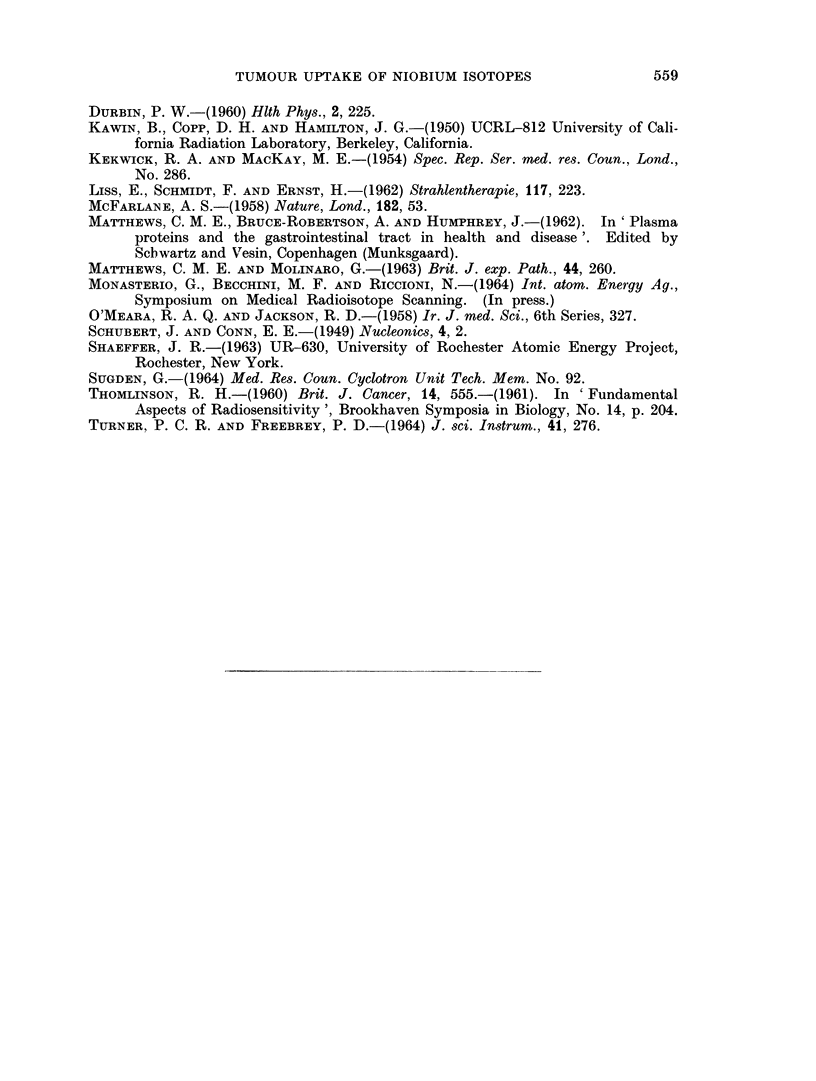# Tumour uptake and distribution of niobium isotopes in rats.

**DOI:** 10.1038/bjc.1965.68

**Published:** 1965-09

**Authors:** C. M. Matthews, J. M. Gartside

## Abstract

**Images:**


### TUMOUR UPTAKE AND DISTRIBUTION OF NIOBIUM ISOTOPES IN RATS

C. M. E. MATTHEWS AND J. M. GARTSIDE

*From the Medical Research Council Cyclotron Unit, Hammersmith Hospital, Ducane Road, London, W.12*

Received for publication February 24, 1965

COMPARISON of the uptake of 17 different radioactive substances by brain and tumour in rats has shown that in these tumours $^{95}$Nb oxalate is taken up in higher concentration than any other isotope investigated (Matthews and Molinaro, 1963). The distribution and metabolism of this isotope in tissues has been further examined in an attempt to improve tumour uptake sufficiently to enable it to be used in diagnosis.

One possibility might be to label fibrinogen with $^{95}$Nb, since there is evidence of increased fibrin concentration in tumours compared with normal tissue (O'Meara and Jackson, 1958). $^{131}$I labelled fibrinogen is said to concentrate in tumours in both humans and animals (Day, Planinsek and Pressman, 1959a, b ; Bale, Spar and Goodland, 1962 ; Liss, Schmidt and Ernst, 1962 ; Shaeffer, 1963 ; Monasterio, Becchini and Riccioni, 1964). The concentration of $^{131}$I radioactivity in tumours, however, may be reduced by deiodination and subsequent equilibration of the $^{131}$I with the iodide pool of the body (Liss *et al.*, 1962). Higher concentrations of radioactivity in tumours might be obtained if fibrinogen could be labelled with a different isotope, for example one which was less soluble or diffused less readily.

It has been concluded that niobium isotopes become bound to plasma proteins because of the observation that the concentration in the blood only decreases slowly after intravenous injection (Kawin, Copp and Hamilton, 1950 ; Durbin, 1960). Since there is evidence that tumours tend to concentrate plasma proteins (Busch, Simbonis, Anderson and Greene, 1956 ; Babson and Winnick, 1954) a possible explanation of the high tumour uptake obtained with niobium isotopes is the binding by plasma proteins. If different plasma proteins could be labelled with $^{95}$Nb it might be possible to influence tumour uptake.

The present paper is an account of a preliminary investigation of the factors affecting the distribution and tumour uptake of niobium isotopes. Although this is not yet fully understood and a satisfactory method of labelling plasma proteins with $^{95}$Nb has not yet been developed, the problems which have arisen may be of interest to other workers. The tumours used were two transplantable sarcomas in rats and it is recognised that human tumours may not be similar. However, for this preliminary work animal experiments are more suitable and the results can be verified later in human tumours.

### METHODS

### *Isotopes*

Two different isotopes of niobium were used, $^{95}$Nb and $^{90}$Nb, both carrier free in a solution of 0·5 % oxalic acid. $^{95}$Nb was obtained from the Radiochemical

Centre, Amersham, or from Oak Ridge, and $^{90}Nb$ was made on the cyclotron in this Unit by bombarding zirconium with deuterons and chemically separating the $^{90}Nb$ (Sugden, 1964). $^{95}Nb$ has a half life of 35 days and emits $\beta$ particles (mainly 0·16 MeV) and $\gamma$ rays of 0·76 MeV. $^{90}Nb$ has a half life of 14·6 hours and emits positrons and $\gamma$ rays of various energies.

*$^{131}I$ HSA*

Human serum albumin (from Lister Institute, fraction AP2, Kekwick and MacKay, 1954) was iodinated by the iodine monochloride method (McFarlane, 1958).

*Animals*

The rats used were of "Johns' Strain", derived from a single pair of Wistar siblings in 1939. Male and female rats from about 200–300 g. were used.

*Tumours*

*RIB*5.—This was a fibrosarcoma which was induced in the strain by Dr. R. Johns by injection of benzopyrene in 1945.

*SBS*1.—This was a moderately well differentiated fibrosarcoma with less necrotic tissue than the RIB5. It developed spontaneously in a rat of the strain in association with congenital spinabifida.

Both tumours were implanted subcutaneously in the flank as described by Thomlinson (1960). By this method tumour cells are made into pellets held together by calcium alginate and the pellets are then tied within the lumen of small pieces of intestine from a young rat. (For the SBS1 the alginate could be omitted). This procedure prevents early metastasis, and produces rounded tumours unattached to skin or muscle whose size can easily be measured externally with calipers. The growth curve of the RIB5 tumour is given by Thomlinson (1961). Tumours were allowed to grow to at least 9 mm. in diameter before they were used.

*Procedure*

Rats were injected intravenously in the tail with a solution of the isotope in up to 2 ml. of physiological saline. The solution of the isotope was prepared in different ways as follows:

*Solution* (*a*).—The stock solution of radioactive niobium in 0·5 % oxalic acid was diluted with saline and the pH was adjusted to 7·4 by adding dilute NaOH, dropwise.

*Solution* (*b*).—The stock solution was simply diluted with saline. In this case the pH was about 1–2.

*Solution* (*c*).—The stock solution was diluted with saline and the pH was adjusted to 4·5.

*Solutions* (*d*).—Attempts were made to label various plasma proteins by either incubating the isotope with the protein solution and then dialysing to remove unbound isotope, or by dialysing the protein solution against saline or citrate buffer containing the isotope. In both cases about 20 hours at room temperature were required to obtain maximum labelling as measured by per cent of radioactivity in trichloracetic acid (TCA) precipitates. However, at 37° C. 2 hours'

incubation was sufficient. Only about 5 per cent of the total radioactivity finally became bound to protein. The proportion of this which was precipitable by TCA could be increased by dialysis. Proteins used included human albumin, rat plasma, and $Na_2SO_4$ fractions of rat plasma proteins. The proportion of $^{95}Nb$ apparently bound was higher with rat plasma than with any of the fractions. Only a few experiments using these protein solutions were satisfactory (see results section), and so the methods are not described in detail.

At different times after injection the rats were anaesthetised with ether, a blood sample was taken from the heart, the animals were killed and dissected, and the organs weighed immediately.

*Measurement of radioactivity*

Radioactivity in rats was measured using a counter consisting of a ring of G26Pb Geiger tubes with removable lead filters for attenuation. Radioactivity in organs was measured by sodium iodide crystal scintillation counting; the large organs were placed between two $1\frac{1}{2}$ in. crystals and the small ones in the well of one of these crystals. Both scintillation and Geiger counters were calibrated for sources of different volumes, using solutions of the isotopes in water and a correction for volume and position was applied to each organ measurement.

*Positron camera pictures*

Pictures of the distribution of radioactivity were taken with a positron camera (Turner and Freebrey, 1964) using $^{90}Nb$. This device, which was invented by Anger (1963), uses a large sodium iodide crystal scintillation counter in coincidence with one or more smaller crystals. The source is placed between the crystals, close to the large one. Pairs of $\gamma$ rays at 180° are produced by annihilation of positrons, and only those pairs that pass through both crystals give rise to pulses which are recorded. The position of the flash of light produced by a $\gamma$ ray in the large crystal is computed electronically from the relative amount of light falling on each of the photomultipliers behind this crystal, and a dot is recorded on the corresponding position on an oscilloscope screen. Thus a dot picture is built up which shows the distribution of isotope in the source. This is photographed and the picture obtained enlarged, so that it can be superimposed on a photograph of the rat.

*Paper electrophoresis*

Paper electrophoresis was carried out by standard methods using Tris buffer at pH 7·6, 8·6 and 9·2, and Whatman No. 3 Chromatography paper.

### RESULTS

When the $^{95}Nb$ solution (a) at pH 7·4 was injected, a high uptake was often found in liver, and this varied considerably in different experiments. A high liver uptake was never found with isotope solution (b) when free oxalic acid was not neutralised, nor with solution (c) when it was partly neutralised to pH 4·5. It seems likely that when all the oxalic acid is neutralised a colloidal solution is formed which is taken up by the reticuloendothelial system. Schubert and Conn (1949) discuss the colloidal properties of some fission products and they find that oxalic acid prevents formation of a colloidal solution of carrier free $^{95}Nb$. After the first few experiments, therefore, the isotope solution was always adjusted to pH 4·5 before injection.

The distribution in organs at different times after injection is shown in Table I for rats with SBS1 tumours. Some results obtained with $^{131}$I labelled human

TABLE I.—*Uptake of Isotope in Rat Organs After Intravenous Injection (% dose/g.)*

| | $^{95}$Nb pH 4·5 | | | | | | | $^{131}$I HSA |
|---|---|---|---|---|---|---|---|---|
| Hours | 1·2 | 6·1 | 24·5 | 30 | 47 | 72·3 | 96·4 | 24·3 |
| Number of rats | 2 | 2 | 10 | 1 | 2 | 3 | 2 | 4 |
| Tumour | 1·43 | 1·76 | 2·07 | 2·15 | 1·74 | 1·42 | 1·31 | 0·690 |
| Plasma | 10·4 | 4·35 | 3·23 | 2·31 | 1·83 | 1·19 | 0·589 | 2·02 |
| Blood | 5·26 | 2·26 | 1·70 | 1·64 | 1·44 | 0·633 | 0·346 | 1·13 |
| Spleen | 0·93 | 1·06 | 2·17 | 2·78 | 1·58 | 3·39 | 3·27 | 0·201 |
| Kidney | 1·29 | 1·05 | 1·76 | 2·51 | 2·29 | 2·60 | 2·73 | 0·306 |
| Liver | 0·517 | 0·517 | 0·489 | 0·745 | 0·533 | 0·583 | 0·583 | 0·226 |
| Lymph nodes | 1·03 | 0·927 | 1·36 | 1·41 | 1·47 | 1·92 | 1·57 | 0·281 |
| Marrow (1 femur) | 1·75 | 2·75 | 1·89 | — | 1·47 | 2·75 | 1·90 | 0·282 |
| Bone + marrow (1 femur) | 0·452 | 0·325 | 1·06 | 1·06 | 0·889 | 1·11 | 1·20 | 0·148 |
| Lungs | 1·53 | 1·44 | 0·777 | 0·878 | 0·657 | 0·683 | 0·925 | 0·704 |
| Intestine | 0·284 | 0·261 | 0·210 | 0·302 | 0·224 | 0·192 | 0·151 | 0·312 |
| Muscle | 0·217 | 0·201 | 0·211 | 0·173 | 0·208 | 0·171 | 0·158 | 0·108 |
| Skin | 0·297 | 0·341 | 0·401 | 0·444 | 0·331 | 0·521 | 0·354 | 0·363 |
| Brain | 0·201 | 0·0950 | 0·0765 | 0·0775 | 0·0504 | 0·0384 | 0·0287 | 0·0456 |
| Testes | 0·467 | 0·618 | 0·761 | 0·955 | 0·818 | 0·614 | 0·782 | 0·267 |
| | Figures expressed as % *dose*. | | | | | | | |
| Blood[1] | 89·6 | 41·0 | 29·4 | 31·2 | 16·5 | 10·8 | 5·07 | 20·5 |
| Muscle[2] | 23·6 | 23·5 | 23·1 | 21·1 | 23·3 | 18·9 | 14·9 | 12·6 |
| Skin | 11·6 | 14·7 | 15·6 | 17·1 | 13·6 | 14·8 | 12·1 | 16·6 |
| Liver | 4·61 | 4·54 | 4·04 | 5·37 | 4·14 | 4·46 | 4·10 | 1·86 |
| Kidneys | 2·07 | 2·38 | 2·88 | 4·00 | 3·53 | 3·99 | 3·89 | 0·553 |
| Intestine | 4·64 | 4·80 | 3·52 | 4·28 | 3·36 | 2·66 | 2·07 | 4·78 |
| Tumour | 1·71 | 1·65 | 2·49 | 2·94 | 2·25 | 2·79 | 2·44 | 1·07 |
| Whole body | 99·5 | 88·7 | 89·2 | 90·8 | 81·1 | 70·3 | 64·5 | 63·8 |

[1] Taking blood volume as 7% body weight.
[2] Taking muscle mass as 45% body weight, (Caster, Poncelet, Simon and Armstrong, 1956).
Radioactivity in organs includes that due to the blood in each organ.

serum albumin are also shown for comparison. The distribution of radioactivity in the different organs is similar for $^{95}$Nb and $^{131}$I HSA, although excretion is less for $^{95}$Nb so that concentrations tend to be higher than for $^{131}$I. In tumour, spleen, kidney, lymph nodes and bone marrow, $^{95}$Nb concentrations are considerably higher than $^{131}$I concentrations. The variation of concentration of radioactivity with time is shown in Fig. 1 for plasma and whole body, and in Fig. 2 for tumour, spleen and kidney. For other organs the concentrations were approximately constant up to about 96 hours, or else fell slightly.

Table II shows tumour uptakes at about 24 hours after injection for SBS1 and RIB5 tumours of different weights. SBS1 tumours probably show some decreased uptake with increasing tumour size. Although there may be a similar effect with RIB5 tumours, there is much greater variation in tumour concentration of niobium.

When solutions (d) were injected, that is, protein solutions in which the $^{95}$Nb was partly precipitable with TCA, high uptakes were sometimes found in liver, spleen or lungs, probably again due to formation of a colloidal solution. In other cases the distribution in the various organs did not differ significantly from results

FIG. 1.—Variation of whole body and plasma $^{95}Nb$ radioactivity with time after intravenous injection.

FIG. 2.—Variation of $^{95}Nb$ concentration in tumour, spleen and kidney with time after intravenous injection.

TABLE II.—*Tumour $^{95}Nb$ Concentrations (pH 4·5) at 24 Hours*

| SBS 1 | | RIB 5 | |
|---|---|---|---|
| Weight | % dose/g. | Weight | % dose/g. |
| 0·678 | 2·32 | 0·96 | 3·28 |
| 0·801 | 1·69 | 1·02 | 1·28 |
| 0·814 | 1·95 | 1·09 | 1·17 |
| 1·08 | 2·08 | 1·51 | 1·50 |
| 1·18 | 2·01 | 1·69 | 4·86 |
| 1·23 | 2·57 | 2·15 | 1·86 |
| 1·31 | 1·88 | 2·70 | 1·88 |
| 1·33 | 2·29 | 4·10 | 1·47 |
| 1·47 | 2·13 | 4·70 | 1·84 |
| 2·15 | 1·87 | 4·77 | 2·21 |
| 3·61 | 1·00 | 4·94 | 1·27 |
| 3·68 | 0·904 | 13·7 | 1·36 |
| 4·57 | 1·710 | | |
| 4·59 | 1·03 | | |
| 5·07 | 1·03 | | |
| 5·41 | 1·05 | | |
| 10·6 | 0·978 | | |

shown in Tables I and II, so that it is doubtful if $^{95}Nb$ was really protein bound. TCA precipitation of plasma samples taken at different times after intraperitoneal injection indicated that from 60 to 80 % of $^{95}Nb$ was protein bound.

Fig. 3 shows a picture of $^{90}Nb$ distribution in a rat with an RIB5 tumour taken with the positron camera. It was found that the tumour could be clearly seen if it contained at least 10 % of the radioactivity in the whole animal ; this corresponds to a tumour of about 15 mm. in diameter.

Paper electrophoresis was carried out in an attempt to investigate which protein binds $^{95}Nb$ but results were inconclusive under the conditions used since $^{95}Nb$ alone moved to the same position as the plasma proteins, and a broad peak of radioactivity was obtained, so that it was impossible to determine whether the isotope was in fact bound to protein or not.

### DISCUSSION

The similar distribution of $^{95}Nb$ and $^{131}I$ labelled plasma proteins indicates that niobium isotopes probably do become protein bound after injection. However, this is difficult to prove conclusively since paper electrophoresis cannot be used, unless a more suitable buffer or type of paper can be found. Also protein precipitation methods may not give reliable results owing to the possible formation of a colloidal solution of $^{95}Nb$ which may be carried down with the protein precipitate. At short intervals after injection much of the radioactivity in organs will be due to their blood content. In an earlier series of experiments this blood radioactivity was subtracted and the extravascular radioactivity was obtained (Matthews and Molinaro, 1963) ; results were similar for $^{95}Nb$ and $^{131}I$ HSA. Most of the extravascular $^{95}Nb$ is in muscle and skin just as for $^{131}I$ labelled albumin (Matthews, Bruce-Robertson and Humphrey, 1962). The plasma radioactivity curve for $^{95}Nb$ is also similar to that obtained with $^{131}I$ labelled plasma proteins (Campbell, Cuthbertson, Matthews and McFarlane, 1956 ; Cohen, 1957),

---

EXPLANATION OF PLATE.

FIG. 3.—Distribution of $^{90}Nb$ in a rat bearing an RIB5 tumour taken with the positron camera.

3

Matthews and Gartside.

and the biological half life is close to that of $^{131}I$ labelled fibrinogen or alpha globulin. The initial fall in plasma radioactivity seems to be rather more rapid with $^{95}Nb$ than with $^{131}I$ proteins but there are too few measurements to indicate whether this is really a significant difference. When plasma samples were fractionated by sodium sulphate precipitation, the TCA precipitable radioactivity was divided between albumin and $\gamma$ globulin fractions ; both these fractions would contain some alpha globulin. The fibrin clot, however, contained very little radioactivity. It seems possible, therefore, that $^{95}Nb$ may become bound to alpha globulin, but more experiments are needed to confirm this.

The $^{95}Nb$ radioactivity which leaves the plasma is not all excreted (Fig. 1) but is partly taken up by the organs, so that organ/plasma concentration ratios increase with time. For most organs the absolute concentration decreases slightly with time, but for kidney and spleen there is a pronounced rise up to 96 hours after injection for kidney and up to about 48 hours for spleen. This rise is roughly parallel to the excretion curve of $^{95}Nb$, as shown in Fig. 4 where spleen and kidney

FIG. 4.—Variation with time after injection of $^{95}Nb$ radioactivity in the kidney and the spleen compared with total radioactivity excreted (100—% dose in body).

uptakes are plotted as percentage dose. This seems to indicate either that after protein catabolism $^{95}$Nb is released in a form which is taken up by spleen and kidney, or possibly that the plasma protein concerned is catabolised in these organs.

Tumour $^{95}$Nb concentration rises during the first 24 hours after injection and then falls. The fall in concentration seems to be merely a reflection of the growth of the tumour since the percentage dose remains approximately constant. The higher 24 hour uptake for some small tumours (Table II) might possibly be due to the effect of inflammation after the transplantation as observed by Day *et al.* (1959b), although the interval between implantation and injection was never less than about 10 days whereas Day *et al.* found increased $^{131}$I fibrinogen uptake due to inflammation for only about 5 days after implantation. For large tumours the uptake is similar for RIB5 and SBS1 tumours.

For investigation of tumour uptake in patients $^{90}$Nb has advantages over $^{95}$Nb since its short half life enables a larger number of mc to be given for the same radiation dose and also pictures of the distribution can be taken with the positron camera. However, if tumour uptake is to be followed for longer times, more $^{90}$Nb must be given and then radiation dose is lower with $^{95}$Nb.

Much more work is required before $^{95}$Nb metabolism and tumour uptake is fully understood and this paper is intended to indicate some of the interesting lines of investigation which may give information about plasma protein metabolism as well as about tumour uptake.

SUMMARY

The distribution of carrier free niobium isotopes in rats and uptake in two different transplanted rat sarcomas is described. Factors affecting this distribution, including binding to plasma proteins, are discussed. The investigation is complicated by the fact that niobium isotopes readily form colloidal solutions.

We would like to thank Dr. R. H. Thomlinson for the rat tumours and for advice, Mr. P. C. R. Turner for the positron camera picture, Professor J. F. Fowler for a digital computer programme used to calculate results, Dr. N. B. Myant for advice with the paper electrophoresis and Mr. D. D. Vonberg for his support and encouragement. We also thank Mr. E. Bamgboye and Miss M. White for much valuable assistance.

### REFERENCES

Anger, H. O.—(1963) *Nucleonics*, **10**, 56.

Babson, A. L. and Winnick, T.—(1954) *Cancer Res.*, **14**, 606.

Bale, W. F., Spar, I. L. and Goodland, R. L.—(1962) ‘ Use of Radioisotopes in Animal Biology and the Medical Sciences ’, **2**, 271, *Int. atom. Energy Ag.* (Academic Press).

Busch, H., Simbonis, S., Anderson, D. C. and Greene, H. S. N.—(1956) *Proc. Am. Ass. Cancer Res.*, **2**, 98.

Campbell, R. M., Cuthbertson, D. P., Matthews, C. M. E. and McFarlane, A. S.—(1956) *Int. J. appl. Radiat. Isotopes*, **1**, 66.

Caster, W. O., Poncelet, J., Simon, A. B. and Armstrong, W. D.—(1956) *Proc. Soc. exp. Biol. Med.*, **91**, 122.

Cohen, S.—(1957) *S. Afr. J. med. Sci.*, **23**, 245.

Day, E. D., Planinsek, J. A. and Pressman, D.—(1959*a*) *J. nat. Cancer Inst.*, **22**, 413.—(1959*b*) *Ibid.*, **23**, 799.

DURBIN, P. W.—(1960) *Hlth Phys.*, **2**, 225.
KAWIN, B., COPP, D. H. AND HAMILTON, J. G.—(1950) UCRL–812 University of California Radiation Laboratory, Berkeley, California.
KEKWICK, R. A. AND MACKAY, M. E.—(1954) *Spec. Rep. Ser. med. res. Coun., Lond.*, No. 286.
LISS, E., SCHMIDT, F. AND ERNST, H.—(1962) *Strahlentherapie*, **117**, 223.
MCFARLANE, A. S.—(1958) *Nature, Lond.*, **182**, 53.
MATTHEWS, C. M. E., BRUCE-ROBERTSON, A. AND HUMPHREY, J.—(1962). In ' Plasma proteins and the gastrointestinal tract in health and disease '. Edited by Schwartz and Vesin, Copenhagen (Munksgaard).
MATTHEWS, C. M. E. AND MOLINARO, G.—(1963) *Brit. J. exp. Path.*, **44**, 260.
MONASTERIO, G., BECCHINI, M. F. AND RICCIONI, N.—(1964) *Int. atom. Energy Ag.*, Symposium on Medical Radioisotope Scanning. (In press.)
O'MEARA, R. A. Q. AND JACKSON, R. D.—(1958) *Ir. J. med. Sci.*, 6th Series, 327.
SCHUBERT, J. AND CONN, E. E.—(1949) *Nucleonics*, **4**, 2.
SHAEFFER, J. R.—(1963) UR–630, University of Rochester Atomic Energy Project, Rochester, New York.
SUGDEN, G.—(1964) *Med. Res. Coun. Cyclotron Unit Tech. Mem.* No. 92.
THOMLINSON, R. H.—(1960) *Brit. J. Cancer*, **14**, 555.—(1961). In ' Fundamental Aspects of Radiosensitivity ', Brookhaven Symposia in Biology, No. 14, p. 204.
TURNER, P. C. R. AND FREEBREY, P. D.—(1964) *J. sci. Instrum.*, **41**, 276.